# Gout in the Throat and Nose

**Published:** 1900-12-29

**Authors:** 


### GOUT IN THE THROAT AND NOSE.

The gouty throat we are all acquainted with. Gout in the nose, however, is probably not so commonly recognised; yet there can be no doubt that if we modify the phrase a little, and speak of the nose conditions which arise in people who are fit subjects for gout, we can all of us point to plenty of examples of such ailments. Dr. Scanes Spicer,[1] writing on the manifestations of gout in the throat and nose, begins by saying that although "regular" gout is characterised by renal inadequacy and consequent uratic accumulation, it appears that this is only one of the terminal or later results of a usually long-standing constitutional condition which need not, and in the majority of cases does not, go on to renal disease or "regular" gout. The exact definition of gout in this enlarged sense is, as he justly adds, a matter of some difficulty. He says, however, that clinically it is easily recognised. "Its subjects are usually those who lead a sedentary life, who eat a great deal more animal food and a great deal oftener than their lack of physical exercise renders necessary or desirable. Hence they develop sooner or later a condition of hyper-alimentation, with deficient combustion of the foodstuffs in the muscles, and deficient elimination of waste-products of tissue vitality by all the emunctories—bowels, kidneys, skin and lungs. Added to this, these patients often indulge to excess—physiologically speaking—in alcohol, which further tends to inhibit tissue change." Perhaps it would be fair to say that these habits and modes of life while they undoubtedly tend to the production of gout, equally tend to produce a long string of maladies among which are certain affections of the throat and nose. Still, as sons of the same father may fairly be called by the same name, and as gout is a simple name and very convenient, let us use it.

Dr. Scanes Spicer says that gout usually manifests itself in the throat as a very chronic catarrh, with frequent subacute exacerbations. The catarrh is characterised by a dull brick-red or purplish coloration, with marked general engorgement, and dilated veins are seen in various positions. The mucous membrane is rough, thickened, often granular, and sometimes glazed. These changes occur on the velum palati, the faucial pillars, and the posterior pharyngeal wall. The tonsils are red and enlarged, and harder than usual, but do not project much. The uvula often shares in the hypertrophy, while the soft palate as a whole is soft and paretic. Behind the posterior pillars, and parallel to them, there are often rough, red, fleshy bands, running from above downwards. The base of the tongue, the epiglottis, &c., are also implicated. Symptomatically this condition is characterised by irritability and hyperæsthesia, abnormal sensations in the throat, retching, especially in the morning while dressing, paroxysmal cough and other unpleasant symptoms, and a special tendency to sub-acute exacerbations.

Gout in the nose is manifested chiefly by a chronic rhinitis of the hypertrophic type. Erectile tumefaction of the membrane, causing recurrent obstruction, takes place on any change of temperature, weather, or bodily position. Thus the nose blocks on lying down, or the obstruction changes from side to side according to the side the patient lies upon, and often the patient is liable to paroxysmal attacks of sneezing on the slightest provocation, and to unpleasant sensations of tickling in the nose and nasopharynx. There is also a tendency to chronic rhinorrhœa, and for the nose to "drip." Subjects of gouty rhinitis are also liable to inflammatory affections of the accessory sinuses. These attacks may pass off without any special treatment, but may form the commencement of a chronic fetid empyema of one or other of the sinuses. Sometimes also the occlusion of the orifices of these sinuses leads to catarrhal conditions of their lining membrane, and thus to so-called gouty headaches, brow agues, and neuralgias, especially such as come on at a given hour every day. One of the commonest results of gouty rhinitis is the habit of mouth breathing and snoring; and then, of course, there is the post-nasal catarrh, the hawking out of thick mucus in the morning, the affections of the ear, and all the other disagreeable consequences of deficient ventilation of the post-nasal space. In the treatment of these gouty conditions of nose and throat Dr. Spicer's experience is overwhelmingly in favour of a visit to a well-arranged spa. Carlsbad and Marienbad must, he says, for some time to come be the ideal spas from the high development and completeness of their arrangements, the natural features of their waters, and their situation and surroundings. Of course many other spas are available, and many of them are in England. Speaking generally, just as much can be done, he says, at an English spa as abroad, under proper supervision, and the patient has the advantage of English medical advice, a shorter journey, and his own language. On the other hand, from the total change of scene, customs, diet, and people, and from being further removed from business cares and worries, some people derive more benefit from a Continental spa. The English *pension* and *table-d'hôte* system also does not compare favourably with the Austrian restaurant system, and is a great drawback to English spas, as its tendency is to allow the habit of overeating to be perpetuated.

[1] Jour. of Balneology and Climatology, Nov., 1900.

